# Case Report: Use of Anakinra in Multisystem Inflammatory Syndrome During COVID-19 Pandemic

**DOI:** 10.3389/fped.2020.624248

**Published:** 2021-02-23

**Authors:** Sara Della Paolera, Erica Valencic, Elisa Piscianz, Valentina Moressa, Alberto Tommasini, Raffaella Sagredini, Valentina Kiren, Manola Comar, Andrea Taddio

**Affiliations:** ^1^Department of Medicine, Surgery and Health Sciences, University of Trieste, Trieste, Italy; ^2^Institute of Maternal and Child Health IRCCS “Burlo Garofolo”, Trieste, Italy

**Keywords:** MISC-C, PIMS-TS, COVID-19, SARS-CoV-2, Kawasaki, anakinra, IL-1

## Abstract

During COVID-19 outbreak, a large number of children with severe inflammatory disease has been reported. This condition, named Pediatric Multi-inflammatory Syndrome temporally associated with COVID-19 (PIMS-TS) or Multisystem Inflammatory Syndrome associated with Coronavirus Disease 2019 (MIS-C), shares some clinical features with Kawasaki disease and is frequently complicated by myocarditis or shock. It has been suggested that MIS-C belongs to the group of cytokine storm syndromes triggered by SARS-CoV-2 infection. So far, intravenous immunoglobulin (IVIG) and systemic glucocorticoids are the most common therapeutic approaches reported in this group of patients. However, the use of anakinra in patients with severe forms of COVID-19 is showing promising results. Here we reported two patients with multisystem inflammatory syndrome complicated with shock. Both the patients presented a poor response to IVIG and systemic glucocorticoids and received anakinra. Treatment with IL-1 receptor antagonist showed a rapid improvement of clinical conditions and biochemical analysis in both patients and demonstrated a good safety profile. Thus, we look forward for future controlled clinical trials with the aim to demonstrate the effectiveness of anakinra in patients with MIS-C and established precise criteria for its use.

## Introduction

Compared to adults, children are less affected by SARS-CoV-2 infection and tend to develop milder forms of the disease (1, 2). However, several children with severe inflammatory conditions, who required intensive care, have been reported during COVID-19 outbreak, especially in those countries with higher incidence of SARS-CoV-2 infection (3–5). There are growing reports of pediatric SARS-CoV-2 related inflammatory conditions, named Pediatric Multi-inflammatory Syndrome temporally associated with COVID-19 (PIMS-TS) or Multisystem Inflammatory Syndrome associated with Coronavirus Disease 2019 (MIS-C), which share some clinical features with Kawasaki disease (KD) and Kawasaki shock syndrome (KSS) (6–8). Clinical characteristics in common with KD are persistence fever, bulbar conjunctivitis, skin rash, or mucosal involvement; however, as the classical criteria of Kawasaki disease may lack in MIS-C, it may be more appropriate to compare this condition to a form of atypical or incomplete Kawasaki disease (9). Moreover, children with MIS- C, compared to classical KD, have an older age and commonly present respiratory and gastrointestinal symptoms; they tend to develop a more severe course of the disease, complicated by cardiac involvement, typically with myocarditis and shock (10–12), and macrophage activation syndrome (MAS) (4, 13, 14). It has been suggested that the syndrome is the result of hyper-inflammation due to a cytokine storm associated with the immune response to SARS-CoV-2 infection (15). In adults with SARS-CoV-2, cytokine storm syndrome has a large spectrum of manifestation and different degrees of severity. Some patients present an inflammatory syndrome with multiorgan dysfunction and severe cytopenia, increased inflammatory markers, hyperferritinemia, and coagulopathy, sometimes meeting the diagnostic criteria for macrophage activation syndrome (15–17). Based on our current knowledge, MIS-C seems a distinct entity but always belonging to the umbrella of cytokine storm syndromes (17, 18).

Anakinra is a recombinant human IL-1 receptor antagonist (IL-1ra) approved for rheumatoid arthritis and other inflammatory conditions (19). Its use showed beneficial effects even in other inflammatory conditions such as severe sepsis in adults (20) or secondary hemophagocytic lymphohistiocytosis (sHLH) in pediatric patients (21). Recent published data reported the effectiveness of anakinra in reducing the risk of death or ICU admittance in patients with COVID-19 (22–24). Case reports and case series in patients with MIS-C reported the treatment with IL-1ra suggesting its safe and effective use in this specific condition (10, 11, 13).

## Case Reports

### Patient 1

**Figure 1:**
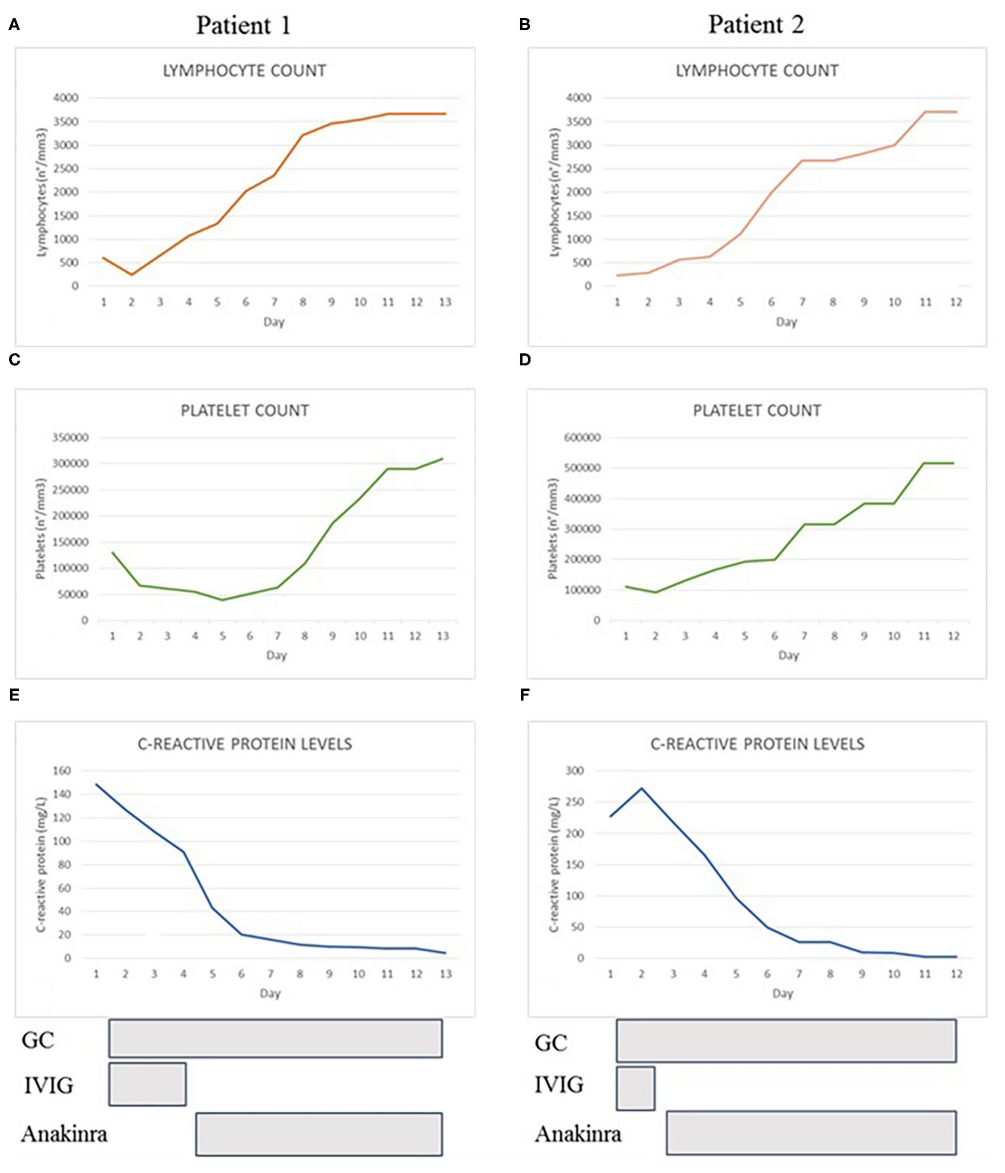
The graphs show the trend of lymphocyte count **(A,B)**, platelet count **(C,D)** and C-reactive protein levels **(E,F)** of both patients during hospitalization. At the bottom we reported the duration and the timing of treatment with glucocorticoids (GC), immunoglobulins (IVIG), and anakinra.

In April 2020, a three-year-old girl of Caribbean ancestry presented to our emergency department for a 2-days history of high fever, abdominal pain and diarrhea. Her medical history reported a close contact with a relative with COVID-19 1 month before, and a recent positivity of naso-pharyngeal swab for SARS-CoV-2. Few hours after admission the patient developed skin rash, bulbar non-exudative conjunctivitis, palmar hands edema and cheilitis. The blood test showed a severe lymphopenia (lymphocyte 250/mm^3^) and thrombocytopenia (platelets 64.000/mm^3^), increased C-reactive protein (CRP 145 mg/L) and liver enzymes (AST 57 U/L, ALT 47 U/L). Coagulation tests showed hypofibrinogenemia (fibrinogen 238 mg/dL), elevation of PT ratio (INR 1.7) and increase of D-dimer levels (4 mg/L). Immunological workup, including immunoglobulin levels and immunophenotypic analysis, was performed to exclude major immune defects. No significant alterations were found in IgG-IgM-IgA levels. Even in the context of a lymphopenia, a normal distribution of lymphocyte subsets like CD27+ memory B cells and, recent thymic emigrants (RTE), together with normal results of perforin expression and NK degranulation and the past medical history of the patient, made unlikely the presence of a severe underlying immune defect (Table 1). Chest X-rays and echocardiography were normal. Bone marrow aspiration highlighted several macrophages with intracytoplasmic vacuoles without sign of hemophagocytosis. Compared with reference values reported by Zauli et al. (25), cytokine levels appeared increased for IL-1Ra (10468 pg/mL), IL-6 (177 pg/mL), IL-10 (363 pg/mL), IP10 (17795 pg/mL), G-CSF (657 pg/mL), MCP1 (299 pg/mL). Treatment with IV methylprednisolone 2 mg/kg/day and intravenous immunoglobulin (IVIG) 2 g/kg was started. After the first dose of IVIG, the patient remained febrile, displaying signs of dyspnea, and shock. She was transferred to our PICU, respiratory support by non-invasive ventilation with CPAP and noradrenaline infusion were started. A thorax CT-scan revealed multiple lung opacities, echocardiography was normal except for a mild mitral valve insufficiency. She received three doses of IVIG without significant improvement, thus we decided to start continuous infusion treatment with anakinra 12 mg/kg/day. Dosage was defined according to a treatment protocol for MAS in children (21). Two days after starting biological therapy, patient clinical conditions gradually improved until defervescence. Treatment with noradrenaline was withdrawn and noninvasive ventilation was stopped. Laboratory workup showed a gradual increase of blood cells count and a reduction of C-reactive protein (Figure 1); a further drop on fibrinogen levels was detected in the first days of therapy and treatment with Low-molecular-weight heparin (LMWH) in addition to fibrinogen was started. Anakinra was gradually tapered and definitely stopped after 8 days. The patient was discharged in good conditions with normal heart function.

**Table 1:** The table shows differences at laboratory tests performed on admission and repeated 1 month after discharge in both patient.

	**Patient 1**		**Patient 2**	
	**At admission**	**At follow-up**	**At admission**	**At follow-up**
Neutrophils (cells/uL)	2030	2670	4770	4400
Lymphocytes (cells/uL)	250	3700	230	1370
Platelets (cells/uL)	64000	335000	112000	236000
Hemoglobin (g/dL)	9	11	10,4	13,9
IgA (mg/dL)	95	146	129	94
IgM (mg/dL)	66	126	58	79
IgG (mg/dL)	749	1196	961	1066
T lymphocytes (cells/uL)	386	2798	150	975
B lymphocytes (cells/uL)	90	579	43	199
NK cells (cells/uL)	44	186	30	176
RTE (cells/uL)	88	594	22	175
CD27+ memory B cells (% of B cells)	7,4	17	12,2	24,2
CD4/CD8 ratio	1,2	0,7	1,3	0,8

### Patient 2

In May 2020, a 10-year-old girl was transferred to our Pediatric Ward for a 5-days history of high fever, abdominal pain with vomiting, headache, maculopapular skin rash and bilateral cervical lymphadenopathy. Patient was previously treated with empiric antibiotics without any improvement. At admission, the patient appeared pale and prostrated. Blood work showed lymphopenia (lymphocyte 230/mm^3^), thrombocytopenia (platelets 93.000/mm^3^), elevation of inflammatory markers (CRP 272 mg/L, ESR 64 mm/h) and ferritin (560 mcg/L). Immunoglobulin levels and immunophenotypic analysis were evaluated to exclude an underlying primary immunodeficiency. No significant alterations were found in IgG-IgM-IgA levels, in main lymphocytes subsets distribution, in CD27+ memory B cells, recent thymic emigrants (RTE), perforin expression and NK degranulation (Table 1). The absence of significant alterations in the distribution of naïve and memory T and B lymphocytes, together with the past medical history of the patient, made unlikely the presence of serious underlying immune defects. Patient 2 suddenly developed tachypnea and hypotension. Echocardiography revealed a dilation of inferior vena cava without heart dysfunction or coronary abnormalities. Thorax CT-scan showed a bilateral ground glass pattern and abdominal ultrasound was normal. Albumin levels were reduced (2.29 g/dL), NT-pro BNP levels were elevated (5,296 pg/mL) and initial coagulation abnormalities were reported (D-dimer 3.6 mg/L, PT ratio 1.6). Naso-pharyngeal swabs were negative for SARS-CoV-2 and other common viral infections. Blood culture was negative. Bone marrow aspiration excluded an hematologic malignancy and a macrophage activation syndrome, whilst it showed several macrophage cells with intracytoplasmic vacuoles. We also measured the patient's inflammatory cytokine levels and found a marked increase of IL-1Ra (1,854 pg/mL), IL-6 (59 pg/mL) and IP10 (11,337 pg/mL) according to reference values reported by Zauli et al. (25). We administered a first dose of IV methylprednisolone 2 mg/kg and IVIG 2 g/kg. After 48 h, clinical conditions and laboratory findings did not improve and subcutaneous anakinra 7 mg/kg/day was started together with antithrombotic prophylaxis with Low-molecular-weight heparin (LMWH). In <24 h, the patient was afebrile, her clinical conditions and laboratory results gradually improved (Figure 1) except for a decrease in fibrinogen levels during the first days after starting the biological treatment. Anakinra was tapered and definitely stopped in 10 days and the patient was discharged. Subsequent clinical, biochemical, and 6-months cardiological follow-up were normal.

## Discussion

We reported two cases of multisystem inflammatory syndrome (MIS-C) diagnosed according to both CDC and WHO criteria and observed during the first wave of SARS-CoV2 epidemic in our pediatrics department. Both of our patients presented some clinical features commonly described in Kawasaki disease (skin rash, cheilitis, bulbar conjunctivitis, cervical lymphadenopathy), they had respiratory and gastrointestinal involvement and rapidly developed shock. Blood work showed marked lymphopenia and thrombocytopenia, increase of inflammatory markers and coagulopathy. Both patients presented several macrophage cells with intracytoplasmic vacuoles at bone marrow aspiration according with the report that COVID-19 infection may induce morphologic and inflammation-related phenotypic changes in peripheral blood monocytes, suggesting that this may be part of the cytokines storm syndrome (26). Only one patient tested positive for the SARS-CoV-2 virus, but most evidence suggests that MIS-C is a delayed inflammatory process to a previous infection; therefore, virus detection can be negative (15). So far, the most common therapeutic approach, reported in large cohorts of patients with MIS-C, consists in intravenous immunoglobulin and systemic glucocorticoids, similarly to patients with KSS. However, recent published data reported a significantly improved clinical outcome in adults with severe form COVID-19 with hyperinflammation treated with anakinra, and a remarkably safe profile of the treatment. Cavalli et al. demonstrated that high-dose anakinra, compared to standard treatment, was associated with a higher survival rate at 21 days, with cumulative survival of 90% in the anakinra group vs. 56% in the standard treatment group, in adults with COVID-19 and a severe hyperinflammatory profile (23). Authors also reported good tolerability of this biological agent, which would control the inflammatory response without affecting viral clearance and without a significant increase of bacterial infections. The results obtained by Cavalli et al. (23) sustained the hypothesis that progression of COVID-19 disease to severe acute respiratory failure is mediated by high levels of circulating proinflammatory cytokines and from these considerations arises the proposal to use anakinra, even early, for the treatment of severe COVID-19 pneumonia. An increasing number of studies on adult population affected by severe COVID-19 pneumonia are demonstrating the effectiveness of IL-1 blockade in reducing oxygen requirement and mechanical invasive ventilation as well as a significantly rapid improvement of patient's clinical conditions and reduction of inflammatory markers (27, 28). Furthermore, an Italian study reported the efficacy of treatment with anakinra as add-on therapy to glucocorticoids in reducing the mortality of adult patients with severe COVID-19 pneumonia and hyperinflammation with a good safety profile (29).

Our experience was in line with these recent evidences. Both our patients improved their clinical conditions after anakinra was started, and no adverse events were reported. We observed a rapid normalization of blood test and a reduction of fibrinogen levels in the first phase of treatment. Fibrinogen is an acute phase reactant promoted by inflammatory cytokines such as IL-1, TNF alfa and IL-6; accordingly IL-1R blockade was reported to decrease fibrinogen levels (30). Thus, while in the first phase of the disease hypofibrinogenemia was secondary to the inflammatory-related coagulopathy, we can assume that further decrease of fibrinogen levels after starting anakinra could be the results of IL-1 blockade. This interpretation is supported by the simultaneous normalization of the remaining coagulation factors and in particular of d-dimer.

Since the beginning of SARS-CoV-2 pandemia four patients with MIS-C were admitted to our pediatric department. Two patients completely recovered with the only use of IVIG and glucocorticoids and did not have any cardiological involvement. The other two patients are those reported in our paper. Their clinical presentation was dramatically more severe and resembled a better-known condition as Kawasaki disease shock Syndrome. A recently published phase II open- label study supported the use of anakinra in patients with IVIG-resistant Kawasaki disease. The authors reported the safety of this drug and its effectiveness in reducing fever, inflammatory markers and coronary artery dilations in patients refractory to at least one dose of IVIG (31). Since anakinra is a promising treatment in this group of patients, it may be considered even in patients with MIS-C with severe cardiological involvement or if lack of response to IVIG and glucocorticoids occurs. Our experience is limited by a small population but during the first phase of pandemia, 53 patients with MIS-C were reported in Italy and anakinra was administered in 4 children who fully recovered without any serious adverse effect. This data is still unpublished but may increase the interest to this treatment strategy.

In addition, clinical and experimental evidence support a crucial role for interleukin-1α in myocardial inflammation and contractile dysfunction after myocardial injury (32, 33). Even for this reason, the use of IL-1 receptor antagonists, such as anakinra, should be considered in patients with MIS-C with cardiologic involvement. Indeed, the central role of IL-1 in the pathogenesis of inflammatory cardiac diseases affirmed the use of anakinra in idiopathic pericarditis (34, 35) and created promising prospective for its use in Kawasaki disease and acute myocarditis (ClinicalTrial.gov NCT02179853, NCT02390596, NCT03018834).

In conclusion, our experience demonstrated the safety of anakinra in children affected by MIS-C who have insufficient response to IVIG and systemic glucocorticoids. Since IVIG and glucocorticoids have already shown effectiveness in large cohorts of patients with MIS-C, we believe this should remain the first line treatment. However, given the risk of rapid evolution to myocarditis or shock (11), we strongly recommend close cardiological observation in children with this severe inflammatory condition. Given the good safety profile of high-dose intravenous anakinra, controlled clinical trials should be driven in order to affirm the effectiveness of this biological agent in a large cohorts of patients with MIS-C and established precise criteria for its use.

## Data Availability Statement

The original contributions presented in the study are included in the article/supplementary materials, further inquiries can be directed to the corresponding author/s.

## Ethics Statement

Ethical review and approval was not required for the study on human participants in accordance with the local legislation and institutional requirements. Written informed consent from the participants' legal guardian/next of kin was not required to participate in this study in accordance with the national legislation and the institutional requirements.

## Author Contributions

SDP wrote the draft. EV and EP did immunological investigations and edited the manuscript. Both ATo and ATa coordinated the study and revised the manuscript. MC, VM, VK, and RS revised the manuscript. All authors contributed to the article and approved the submitted version.

## Conflict of Interest

The authors declare that the research was conducted in the absence of any commercial or financial relationships that could be construed as a potential conflict of interest.
